# Citizen science reveals waterfowl responses to extreme winter weather

**DOI:** 10.1111/gcb.16288

**Published:** 2022-06-16

**Authors:** Nicholas M. Masto, Orin J. Robinson, Michael G. Brasher, Allison C. Keever, Abigail G. Blake‐Bradshaw, Cory J. Highway, Jamie C. Feddersen, Heath M. Hagy, Douglas C. Osborne, Daniel L. Combs, Bradley S. Cohen

**Affiliations:** ^1^ College of Arts and Sciences Tennessee Technological University Cookeville Tennessee USA; ^2^ Cornell Lab of Ornithology Cornell University Ithaca New York USA; ^3^ Ducks Unlimited Inc. Memphis Tennessee USA; ^4^ Division of Wildlife and Forestry Tennessee Wildlife Resources Agency Nashville Tennessee USA; ^5^ National Wildlife Refuge System U.S. Fish and Wildlife Service Stanton Tennessee USA; ^6^ College of Forestry, Agriculture, and Natural Resources University of Arkansas at Monticello Monticello Arkansas USA

**Keywords:** citizen science, climate change, distributions, eBird, extreme climatic event, GPS telemetry, polar vortex, waterfowl, weather severity

## Abstract

Global climate change is increasing the frequency and severity of extreme climatic events (ECEs) which may be especially detrimental during late‐winter when many species are surviving on scarce resources. However, monitoring animal populations relative to ECEs is logistically challenging. Crowd‐sourced datasets may provide opportunity to monitor species' responses to short‐term chance phenomena such as ECEs. We used 14 years of eBird—a global citizen science initiative—to examine distribution changes for seven wintering waterfowl species across North America in response to recent extreme winter polar vortex disruptions. To validate inferences from eBird, we compared eBird distribution changes against locational data from 362 GPS‐tagged Mallards (*Anas platyrhynchos*) in the Mississippi Flyway. Distributional shifts between eBird and GPS‐tagged Mallards were similar following an ECE in February 2021. In general, the ECE affected continental waterfowl population distributions; however, responses were variable across species and flyways. Waterfowl distributions tended to stay near wintering latitudes or moved north at lesser distances compared with non‐ECE years, suggesting preparedness for spring migration was a stronger “pull” than extreme weather was a “push” pressure. Surprisingly, larger‐bodied waterfowl with grubbing foraging strategies (i.e., geese) delayed their northward range shift during ECE years, whereas smaller‐bodied ducks were less affected. Lastly, wetland obligate species shifted southward during ECE years. Collectively, these results suggest specialized foraging strategies likely related to resource limitations, but not body size, necessitate movement from extreme late‐winter weather in waterfowl. Our results demonstrate eBird's potential to monitor population‐level effects of weather events, especially severe ECEs. eBird and other crowd‐sourced datasets can be valuable to identify species which are adaptable or vulnerable to ECEs and thus, begin to inform conservation policy and management to combat negative effects of global climate change.

## Graphical abstract

Extreme climatic events (ECEs) are occurring more often and later in winter which may have negative effects on migratory birds. However, monitoring birds before, during, and after ECEs is challenging. We used 14 years of eBird, a global citizen science initiative, to examine distribution shifts for seven waterfowl species. eBird predicted distribution shifts which we validated using telemetry data from GPS‐tagged mallards. Overall, waterfowl delayed northward migrations in response to late‐winter ECEs. Species with specialized foraging strategies were more affected than generalists. Our study demonstrated eBird's utility as a promising tool to measure climate sensitivity or resilience in migratory waterfowl.

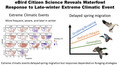

## 1 INTRODUCTION

Global climate change is forecast to increase severity and frequency of short‐term extreme climatic events (hereafter, ECEs; Coumou & Rahmstorf, 2012; IPPC, 2021; Smith, 2011). Extreme climatic events are defined as unusually severe meteorological anomalies (Smith, 2011). For example, ECEs include abnormally severe precipitation, flooding, drought, heatwaves, polar vortex disruptions, and other natural disasters. Extreme climatic events create resource bottleneck conditions which force species to abruptly move, redistribute, or alter other behaviors to survive, ultimately affecting abundance and distribution of wildlife populations (Bailey et al., 2019; Maron et al., 2015). Extreme climatic events also may be as important in understanding ecological response to climate change as long‐term species‐climate trends (Bailey & van de Pol, 2016). Thus, quantifying population responses and identifying vulnerability or resilience to ECEs is and will continue to be critically important for prioritizing future conservation efforts and mitigating negative effects of global climate change on wildlife populations (Martinuzzi et al., 2016; van de Pol et al., 2010).

Drought and polar vortex disruption ECEs drastically alter ambient temperatures and resource availability. Temperature is a critical component of a species' fundamental niche (Magnuson et al., 1979). Temperature affects daily and annual behavior cycles, and a species' thermotolerance influences its seasonal distributions (Londe et al., 2021; Rakowski et al., 2019; Sunday et al., 2012). Likewise, thermoregulation is energetically expensive and can comprise considerable amounts of daily energetic expenditures in birds (e.g., up to 80% in wintering waterfowl; McKinney & McWilliams, 2005). Birds employ a variety of strategies to ameliorate costs associated with thermoregulation such as limiting space‐use (Tanner et al., 2017), selecting thermal refugia (Jorde et al., 1984; Londe et al., 2021), or altering foraging strategies and activity budgets (Brodsky & Weatherhead, 1985). However, if thermoregulatory costs are too great, a viable strategy for mobile taxa such as birds is to move from extreme temperatures or otherwise suffer temperature‐induced mortalities (Doherty & Grubb, 2002; Zuckerberg et al., 2011). In addition to affecting physiological status, temperature and snow cover also may limit food and space availability (Notaro et al., 2016). Consequently, migratory bird populations often shift as a result of temperature‐driven resource depression. Ultimately, species' thermotolerance and population‐level responses to extreme temperatures can directly or indirectly influence fitness (Newton, 2007; Schou et al., 2021). Furthermore, extreme winter weather, particularly during late winter and into early spring, may exaggerate these threats because wintering bird populations are already operating on scarce food resources and many are at the limits of their thermotolerance (Cohen et al., 2021; Newton, 2007).

Four winter storms had widespread impacts across the mid‐continental United States between 11 and 19 February 2021, bringing record freezing temperatures to much of the Southeast, Midwest, and Great Plains (Doss‐Gollin et al., 2021; Erdman, 2021). Given the otherwise mild winter temperatures during the same year, waterfowl biologists speculated that ducks and geese would react to late‐season freezing temperature extremes. Many wintering waterfowl species are resilient to cold temperatures and either become sedentary or increase foraging activities to reduce or offset thermoregulatory energy expenditures (Brodsky & Weatherhead, 1985; Jorde et al., 1984). However, persistent freezing temperatures cause wetlands to freeze, resulting in food and usable space limitations for even the most cold‐tolerant species. Consequently, waterfowl are typically evicted to more southerly locations during such events (Notaro et al., 2016; Schummer et al., 2010). During late‐February, however, most species of waterfowl are experiencing endogenous photoperiodic cue which physiologically prompts fuel deposition and preparedness for spring migration (Gwinner, 2008; Heitmeyer, 1988; McLandress & Raveling, 1981). The push‐pull hypothesis suggests that movement of organisms outside of their core range can be caused by “push” factors (e.g., poor conditions within core range) or “pull” factors (e.g., better conditions elsewhere; Bateman et al., 2015; Fuguitt, 1959). In waterfowl for example, more productive wetlands with greater cover attract (i.e., “pull”) individuals during molt migration (Fox & Walsh, 2012; Fox et al., 2014). Conversely, extreme weather often is considered a “push” pressure that forces movement out of an area (Smith, 1970; van Wijk et al., 2012). The mid‐February 2021 ECE presented an opportunity to examine whether wintering waterfowl populations were “pushed” farther south, potentially delaying spring migration phenology, or experienced stronger “pull” by photoperiod cues for northward migration and stayed at their chosen wintering latitudes at the risk of possible mortality or compromised body condition from extreme temperatures and snowfall.

Documenting waterfowl and other bird responses to climate extremes is difficult because of the opportunistic nature in which these studies need to be conducted (Smith, 2011). Furthermore, the potential for bird responses to span vast areas often precludes observations of them before and after extreme events (Cox Jr. & Afton, 2000). Researchers thus rely on existing data or incidental studies that are typically conducted at small spatial scales (e.g., Bailey et al., 2019); however, ECEs—especially extreme temperatures—typically occur at much larger spatial scales where data are unavailable or sparse to track responses in real time. Fortunately, new and evolving information science and other technologies are enhancing our ability to monitor bird populations at large spatial scales (Sullivan et al., 2009). In particular, citizen‐based observation networks, such as Cornell Lab of Ornithology's eBird project, are globally extensive and continuous data‐streams that are relatively inexpensive to maintain (Sullivan et al., 2014). These citizen‐science databases have previously been mined to examine long‐term temporal trends in bird occupancy, phenology, and species distributions (e.g., across breeding or wintering seasons; Johnston et al., 2021; Mayor et al., 2017; Meehan et al., 2021). However, their utility for studying behavioral phenomena over shorter time intervals (i.e., weeks) has received far less attention. eBird amasses large amounts of data with extremely high spatiotemporal resolutions (La Sorte & Somveille, 2019) and is semi‐structured, collecting many effort variables so that users may account for observation biases inherent in citizen science data (Johnston et al., 2021; La Sorte & Somveille, 2019). Therefore, eBird may provide opportunities to monitor real‐time avian population responses relative to increasingly frequent ECEs (Cohen et al., 2020).

Citizen scientists using eBird continuously collect high resolution occurrence and abundance data before, during, and after extreme weather events. Thus, eBird naturally provides a before‐after experimental setting to examine bird responses to unplanned phenomena such as ECEs. Our goal was to use eBird to examine changes in continental distributions of seven waterfowl species (Anatidae) in response to the February 2021 and other continentally‐distributed extreme late‐winter weather events. We compared changes in waterfowl distributions in 2021 to those observed in years 2008–2020 (excluding years 2015 and 2019), using the latter series of years as reference for years not characterized by an ECE. We sought to verify distributional changes because eBird data rarely are used at such short‐time intervals (weekly), and accuracy is generally assumed but not known. As verification, we compared distribution patterns from eBird data to concurrently available data from individually GPS‐marked Mallards (*Anas platyrhynchos*) during 2020 and 2021 winter periods.

We predicted no significant shifts or minor northward shifts in waterfowl distributions in non‐ECE years because most waterfowl species are preparing for spring migration and early migrators may shift population trajectories in northward directions. Conversely, we predicted waterfowl distributions to shift south in response to ECEs with largest shifts in the Central and Mississippi Flyways where the ECE had greatest impacts. We predicted more pronounced shifts for smaller‐bodied species because of lower thermal inertia compared with larger‐bodied waterfowl (Albright et al., 2017; Huey et al., 2012). Lastly, we expected waterfowl with specialized foraging strategies, such as wetland obligate species (e.g., Northern Shoveler [*Spatula clypeata*]), were disproportionately affected by the ECE because their foraging strategies necessitate open water wetlands unlike other ducks and geese which are able to feed in dry habitats (Baldassarre, 2014).

## 2 METHODS

### 2.1 Extreme weather event

**FIGURE 1:**
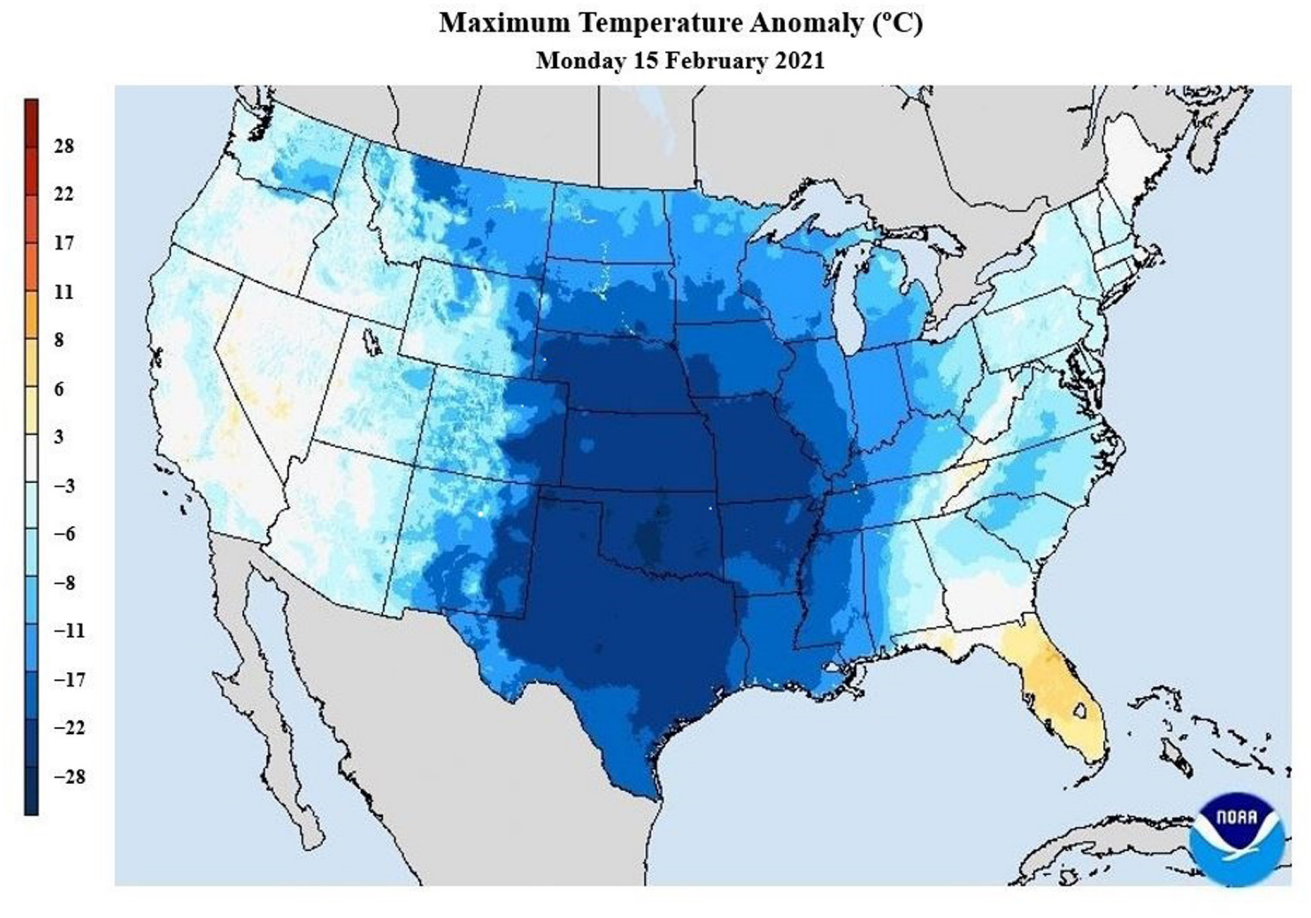
Temperature anomalies (°C) on February 15, 2021 across the conterminous United States. Data used to produce the map are daily locational temperatures from the unrestricted mesoscale analysis (URMA) by National Weather Service of the National Oceanic and Atmospheric Administration. Map credit is owed to the National Weather Service Weather Prediction Center 2021.

Winter storms Shirly, Tabitha, Uri, and Viola blanketed the Midwest and Southeastern U.S. between February 7 and 20, 2021 (13 days). Peak cold temperatures occurred during February 13 through 16, 2021 (Figure 1) resulting from a polar vortex disruption that produced record‐breaking low temperatures in 30% of all U.S. weather stations across 12 mid‐continental states. Notable all‐time low temperatures included Bottineau, North Dakota (−47°C), Owen, Wisconsin (−43°C), Spearfish, South Dakota (−36°C), Sioux City, Iowa (−33°C), Lawton, Oklahoma (−24°C), and Tyler, Texas (−21°C). Air temperatures averaged 22–28°C colder than long‐term averages across much of the Great Plains and mid‐south with temperatures recorded as low as −29°C at latitudes including southern Arkansas and the Texas panhandle. Winter storms Uri and Viola dropped significant snowfall and resulted in the most widespread snowcover across the conterminous United States in nearly two decades (Erdman, 2021). Record snowfall also was recorded in many southern states, with portions of Arkansas receiving >51 cm of snow and Del Rio, Texas receiving 28 cm.

In Memphis, Tennessee (35.1298°, −89.8037°)—the approximate center of our GPS telemetry studies—average temperatures during the storm were −11.4°C (range = −20.6 to −3.9°C). During the same time, Memphis received 25.4 cm snow and >2.54 cm snow remained on the ground during February 15–21. Average daily air temperatures were <0°C between February 12 and 21, leading to extensive ice cover on wetlands, especially those lacking flowing water, during and following the storms. As a comparison, average temperature at the same weather station during February 13–17, 2020 was 4°C and with no snowfall during the entire month (National Centers for Environmental Information, 2021; ncdc.noaa.gov; Accessed March 30, 2021).

### 2.2 eBird data selection and processing

Complete eBird checklists are semi‐structured and geographically referenced lists of occurrence and abundance of every species of bird the citizen scientist is able to identify, and is recorded year‐round and around the world (Sullivan et al., 2014). We downloaded and processed the eBird Basic Data for observations of Mallard, Green‐winged Teal (hereafter, Teal; *A*. *crecca*), Gadwall (*Mareca strepera*), Northern Pintail (*A. acuta*), Northern Shoveler, Greater White‐fronted Goose (*Anser albifrons*), and Snow Goose (*Chen caerulescens*) across North America using the *auk* package in R version 4.0.3 (R Core Team, 2021; Strimas‐Mackey et al., 2018). We limited our selection of eBird checklists to those collected during January 1 to February 28, 2008 through 2021. We used a recommended structured data filtering process outlined in Johnston et al. (2021) to address probable spatial and effort‐based biases associated with the detection process by citizen science observers. Specifically, we (1) selected only “complete” eBird checklists with stationary protocols, or checklists that reported traveling protocols when observers travelled <5 km; (2) filtered observations by unique latitude, longitude, and date combinations to minimize site selection bias and to ensure highly‐surveyed locations were not over‐represented in our datasets; and (3) excluded checklists with >10 observers, excluded duplicate checklists, and limited checklists to ≤5 h duration (Johnston et al., 2021). We acknowledge inaccuracies associated with eBird crowd‐sourced data. However, our filtering process has been shown to effectively reduce inherent biases associated with eBird data (Johnston et al., 2021). For example, recent analyses reported 89% of observed bird locations were ≤1 km from the reported eBird GPS centroid and 86% of search efforts were within 1.5 km radius of the eBird GPS centroid (Cohen et al., 2020). Thus, we eliminated largest potential inaccuracies simply by excluding traveling checklists >5 km.

### 2.3 Animal capture and GPS telemetry

**FIGURE 2:**
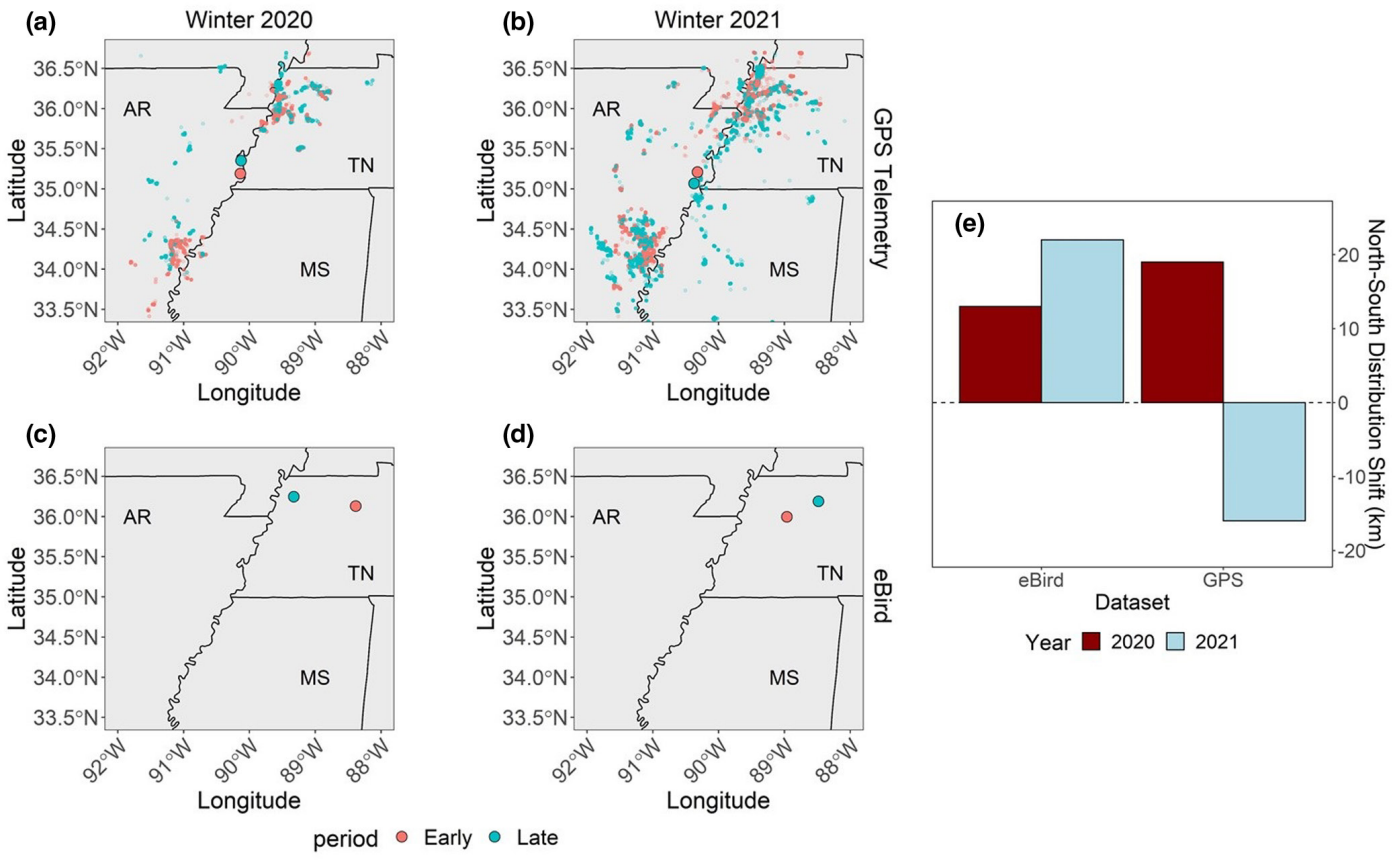
Changes in distributional centers between “early” (January1 to February6; red) and “late” (February7–28; blue) periods during 2020 (column 1; a,c) and 2021 (column 2; b,d) for Mallards using GPS telemetry (row 1; a,b) and eBird data (row 2; c,d). We bounded eBird analysis between 32.5° N and 37.5° N latitudes in the Mississippi Flyway for this comparison. eBird predicted distributional shifts within 6 km of GPS‐marked Mallards in 2020 and within 38 km in 2021 (right panel bar graph).

We captured adult and juvenile male and female Mallards using a combination of swim‐in traps, confusion traps, and rocket‐nets at four locations in Arkansas and eight locations in Tennessee from November to February 2020 and 2021 (Figure 2; Dieter et al., 2009; Sharp & Smith, 1986). We banded all ducks with USGS standard aluminum tarsal bands. We attached 15–20 g solar rechargeable and remotely programable, OrniTrack Global Positioning System‐Global System for Mobile transmitters (GPS‐GSM; Ornitela, UAB Švitrigailos, Vilnius, Lithuania) to birds weighing ≥1000 g (i.e., ≤2.5% of total body mass) to ensure that deployment package remained below recommended body weight limits (3–5%; Fair et al., 2010). We attached transmitters via dorsally mounted body harnesses made of automotive moisture‐wicking elastic ribbon (McDuie et al., 2019). Completed harnesses had two body loops which were knotted and sealed with cyanoacrylic glue above the keel and across the abdomen of the bird (McDuie et al., 2019). Total package of GPS‐GSM transmitter and harnesses at the time of deployment weighed 17–22 g. The GPS‐GSM transmitters were programed to record locations hourly at >75% battery, every 2 h when battery was 25–75%, and every 6 h at <25% battery life. All capture and handling procedures of ducks are in accordance with Tennessee Technological University's Institutional Animal Care and Use Committee (IACUC) protocol #19–20‐002 and University of Arkansas Monticello's IACUC protocol #05172021, and authorized under Federal Banding Permits #05796 and #23825, respectively.

### 2.4 Modeling waterfowl distribution shifts pre‐ and post‐extreme climatic event in 2021

We subset eBird data geographically into the Atlantic, Mississippi, Central, and Pacific Flyways based on U.S. Fish and Wildlife Service administrative flyway boundaries (U.S. Fish and Wildlife Service, 2021; fws.gov/birds/management/flyways; Accessed June 1, 2021). We split these data further into two time periods: January 1 to February 6 (“early”) and February 7 to February 28 (“late”) across all years (2008–2021). The “early” period represented population distributions before the February 2021 ECE and the “late” period represented distributions during and after the ECE. Additionally, we geographically subset eBird data for Mallards in the Mississippi Flyway during the same time periods between latitudes 32.5° and 37.5°N because these latitudes better aligned with Mallard GPS telemetry studies. For each species, we calculated mean positional centroids of eBird observations >0, weighted by their abundance, to determine the center of mass for each period, year, and flyway combination. Similar center of mass calculations have been used to examine shifts in mean wintering distributions of avifauna using crowd‐sourced data (i.e., USGS, Breeding Bird Survey; La Sorte & Thompson III, 2007).

We compared inferences from center of mass analyses from eBird data to the GPS locational data using the same analyses. We estimated positional centroids of GPS‐derived Mallard locations similar to the eBird dataset. Specifically, we subset GPS telemetry locations of Mallards into the same “early” and “late” periods. “Early” and “late” positional centroids across Tennessee and Arkansas were calculated as the mean of all GPS locations, weighted by frequency of locations per state, during “early” and “late” time periods in 2020 and 2021 (Figure 2).

We modeled shifts in distributional centers as the distance (km) between “early” and “late” mean positional centroids (i.e., distance between mean distributional centers [∆]) across the continent and for each flyway separately. We predicted that body size and foraging strategies were important factors associated with species responses to the February 2021 ECE and other severe cold February temperatures. Therefore, we grouped waterfowl species into three foraging strategies: (1) generalists, (2) grubbing foragers, and (3) wetland obligate species. Generalists (i.e., Mallard, Northern Pintail, and Teal) were medium‐sized ducks, with the exception of Teal. Lesser Snow and Greater White‐fronted Geese were categorized as exhibiting grubbing and/or browsing foraging strategies; consequently, “grubbers” were exclusively large‐bodied waterfowl. Lastly, we categorized Northern Shoveler and Gadwall as small‐bodied wetland obligate foragers because their diets are principally based on open water wetland‐dependent invertebrates and vegetation (Euliss et al., 1991). We removed data associated with February 2015 and 2019 because these month/year combinations were characterized by abnormally cold temperatures and we desired to only model effects of the February 2021 ECE (Figure S1). Model structure was:

(1) $$∆\text{distance}\;{\left(\mathrm{km}\right)}_{i,j}\sim \left(\beta_{0}+\alpha_{\text{year}}\right)+\beta_{\text{foraging}\_\text{strategiesi},j}*\beta_{\text{ECEi},j}+\varepsilon_{i,j}\;$$

where distribution shifts (i.e., ∆distance) for observation *i* at spatial scale *j* (i.e., continent or individual flyway) was a function of additive effects (β) of a species' foraging strategy (i.e., generalists, wetland obligate, or grubbing), whether an extreme climatic event occurred or not in a given year, and their interaction.$\;\beta_{0}$ represented the intercept with reference variables of a generalist in non‐ECE years; $\alpha_{\text{year}}$ depicts random intercepts for each year of the study excluding 2015 and 2019; and $\varepsilon_{i,j}$ is residual error for each model (i.e., 4 flyway + 1 continental model; Table S1).

### 2.5 Effect of severe February temperature on continental waterfowl distributions

Lastly, we modeled how waterfowl distributions were affected by severely cold temperatures in February, which we defined as temperature anomalies ≤−4°C across >15% of the conterminous United States (Figure S1). Model structure was similar to the February 2021 ECE analysis; the only difference was the severe February temperatures now included February 2021, 2019, and 2015. We did not fit flyway‐specific models for this analysis because severe cold temperatures occurred at different spatial and temporal scales (Figure S1).

All models were fit in a Bayesian framework in the *rstanarm* package in R (Goodrich et al., 2020; R Core Team, 2021). We specified normally distributed vague priors (μ = 0 and σ = 2.5), and used 3 Markov Monte Carlo chains with 10,000 iterations, 5000 burn‐in iterations, and a thinning interval of 1. We confirmed convergence by visually examining traceplots and Gelman–Rubin statistics with $\hat{R}$ ≤ 1.10 (Brooks & Gelman, 1998). We used the *emmeans* package in R to estimate marginal medians among categorical predictors and their interactions (Lenth et al., 2022; R Core Team, 2021). We present medians with 95% credible intervals in text and posterior distributions with 66 and 95% credible intervals for visual interpretation in supplementary material (Table S1; Figure [Link], [Link]).

## 3 RESULTS

### 3.1 eBird and GPS‐based comparisons of Mallards in the Mississippi Flyway

We GPS‐marked 15 and 53 Mallards in Arkansas and Tennessee, respectively, in autumn–winter 2020. In 2021, we GPS‐marked 84 and 210 Mallards in Arkansas and Tennessee, respectively. From the eBird dataset, we obtained 1794 non‐zero observations for Mallards in 2020 and 2734 in 2021. Mean distributional centers of Mallard GPS locations shifted northward 19 km between “early” and “late” periods in 2020 (Figure 2a). Conversely, mean distributional centers of GPS‐marked Mallard locations shifted 16 km southward between the two periods in 2021 (Figure 2b). For the eBird dataset, mean distributional centers of Mallards shifted northward 13 km between “early” and “late” time periods in 2020 (Figure 2c). Mean distributional centers also shifted northward 22 km in 2021 (Figure 2d). Thus, eBird predicted distributional shifts within 6 km of GPS‐marked Mallards in 2020 and within 38 km in 2021.

### 3.2 Effect of February 2021 ECE on continental waterfowl distributions

**FIGURE 3:**
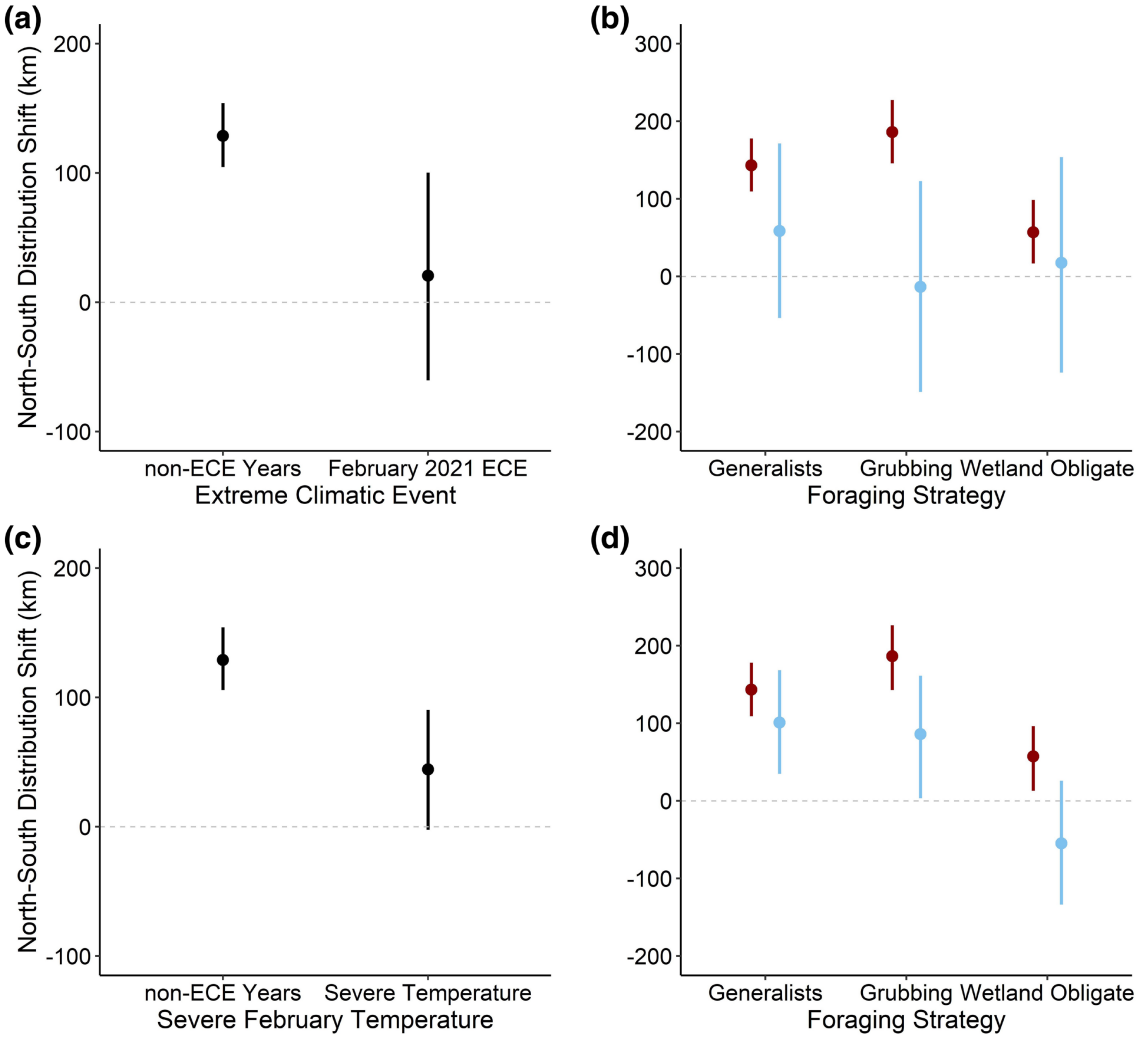
North–south median distribution shifts (km) and 95% credible intervals (CRI) for continental waterfowl populations in response to the February 2021 ECE (a) and severe February temperatures in 2021, 2019, and 2015 (c) compared with non‐ECE years. Estimated marginal median distribution shifts (km) and 95% CRI among waterfowl foraging strategies in response to the February 2021 ECE (b; blue) and severe February temperatures (d; blue) compared with non‐ECE years (red). Foraging strategies included generalists (i.e., Mallard, Northern Pintail, and Teal), grubbing/browsing foragers (i.e., Lesser Snow Goose, and White‐fronted Goose), and wetland obligate species (Northern Shoveler and Gadwall).

In general, continental waterfowl distributions still moved northward between “early” and “late” periods despite the February 2021 ECE, but to a lesser magnitude compared with non‐ECE years (Table S1 and Figure S2). Specifically, across waterfowl taxa, foraging strategies, and flyways, we found northward distribution shifts were 6 times greater in years not characterized by the ECE (128.6 km; 95% CRI = 104.5–154.0 km) compared with distribution shifts in February 2021 (20.6 km; 95% CRI = −60.4–100.0 km; Figure 3a). Flyway‐scale analyses revealed similar trends (Figure S3 and S4). Waterfowl distributions in the Atlantic Flyway shifted 79.1 km northward in non‐ECE years (95% CRI = 34.1–124.0 km) but 42.8 km southward in February 2021 (95% CRI = −191.9–103.0 km). Waterfowl distributions in the Mississippi Flyway shifted 230.0 km northward in non‐ECE years (95% CRI = 182.0–279.0 km) compared with 70.4 km in the 2021 ECE year (95% CRI = −102.0–228.0 km). A similar trend was present in the Central Flyway where northward shifts were 2.5 times greater in non‐ECE years compared with February 2021 (i.e., 161.2 km [95% CRI = 99.3–219 km] and 63.2 [95% CRI = −139.3–254 km], respectively). Waterfowl distributions remained approximately constant in the Pacific Flyway, shifting 44.8 km northward in non‐ECE years (95% CRI = 8.6–83.5 km) compared with 9.5 km southward in the 2021 ECE year (95% CRI = −138.7–109.0 km; Figure S4).

Model results suggested no clear interactive effects between the February 2021 ECE and waterfowl foraging strategies (i.e., generalist, grubbing/browsing, and wetland obligate species), especially when data were subset by flyways (Figure 3). Nevertheless, estimated marginal medians suggested that waterfowl with grubbing foraging strategies (i.e., geese) may have been more sensitive to the February 2021 ECE (Figure 3c). Grubbing forager distributions shifted northward 185.8 km in non‐ECE years (95% CRI = 145.7–227.3 km) but southward 13.7 km in February 2021 (95% CRI = −149.1–122.9 km). Generalists' distributions (i.e., Mallard, Northern Pintail, Teal) shifted 58.5 km northward in February 2021 (95% CRI = −149.1–122.9 km), approximately half the distance northward compared with non‐ECE years (143.1 km; 95% CRI = 109.2–177.6). Wetland obligate species distributions shifted 56.8 km northward in non‐ECE years (95% CRI = −52.8–93.5 km) and 17.3 km southward in February 2021 (95% CRI = −124.1–154.7 km). Within flyways, no clear effect of the February 2021 ECE relative to different foraging strategies was apparent. However, nearly all estimated northward distribution shifts among foraging strategies were greater in reference years compared with the 2021 ECE year (Figure 3).

### 3.3 Effect of severe February temperature on continental waterfowl distributions

Across waterfowl taxa and flyways, severe February temperatures shortened northward distribution shifts by 84.8 km (95% CRI = 33.4–138.0) compared with years characterized by normal, milder weather (Figure 3c). The effect of severe temperatures was exaggerated among specialized foragers (Figure 3d). In particular, grubbing and browsing waterfowl (i.e., geese) moved 186.4 km northward in milder non‐ECE years (95% CRI = 142.73–226.2 km) compared with only 86.0 km during severe Februarys (95% CRI = 3.18–161.2 km; Figure 3d). Additionally, wetland obligate foragers shifted 57.2 km northward in milder non‐ECE years (95% CRI = 13.0–96.2 km), compared with 54.9 km in the opposite, southward direction, in response to delayed and severe weather in February (95% CRI = −133.9–25.9 km; Figure 3d). However, generalists did not appear to be affected, shifting their distributions northward 143.3 km in non‐ECE years (95% CRI = 109.0–178.0 km) versus 101.0 km in severe weather years (95% CRI = 34.67–168.3 km).

## 4 DISCUSSION

Crowd‐sourced science has informed ecology and conservation (e.g., Ruiz‐Gutierrez et al., 2021), primarily because of increasing participation and data collection at broad spatiotemporal scales (Chandler et al., 2017; Pocock et al., 2017). Despite increasing participation, few studies have evaluated the utility of eBird data to monitor bird populations at daily–weekly time intervals (Cohen et al., 2020, 2021; Neate‐Clegg et al., 2020), which are time intervals when species cope with ECEs. Our comparison of eBird and GPS‐based center of mass distributions for Mississippi Flyway Mallards before and after the February 2021 ECE suggested eBird may be useful to detect species distribution shifts at short time intervals, depending on needed spatial resolution. After cautious filtering (sensu Johnston et al., 2021), other studies also have found adequate performance of citizen science datasets relative to those that were scientifically collected (Coxen et al., 2017; Jackson et al., 2015; Lin et al., 2015). More recent studies used eBird to predict short‐term responses (i.e., 5 days–2 weeks) to ECEs and also reported adequate performance for many species, especially abundant ones like waterfowl (Cohen et al., 2020, 2021). Therefore, while we acknowledge possible, yet unknown bias in eBird distributions in our analyses, our results suggest eBird can be used to monitor waterfowl and other avian distributions in response to ECEs as well as other short‐term events. Additionally, we believe eBird is most useful for monitoring avian responses during short timespans when applied across greater spatial extents. Limiting spatial bounds between 32.5 and 37.5°N in the Mississippi Flyway for our comparative analysis of Mallards reduced our eBird sample size by 94%. eBird datasets will become increasingly useful at smaller spatial scales as participation continues to grow.

Our prediction that waterfowl species' distributions shifted southward in response to the February 2021 polar vortex disruption and that southern shifts would be most apparent where temperature anomalies were greatest (i.e., Central and Mississippi Flyways) was not supported. Northern—not southern—distribution shifts were most common across all species in the Mississippi and Central Flyways, but they were approximately half the northward distance in 2021 compared with non‐ECE years. Thus, waterfowl were affected by the ECE but not the way we anticipated. Instead of southern distribution shifts, waterfowl stalled northern migrations until the ECE was over and were likely able to do so because of physiological and behavioral responses to deal with extreme cold temperatures in the short term. Previous research has demonstrated that severe weather “pushes” dabbling ducks south in autumn–winter (Notaro et al., 2016; Schummer et al., 2010). Indeed, we speculate that would the ECE have occurred in December rather than February, we may have seen more southward distribution shifts across all waterfowl species as predicted (Notaro et al., 2016). However, it is likely that the timing of the ECE mitigated its effect on shifting waterfowl distributions and suggests photoperiodic cues to begin spring migration must have been a stronger “pull” overall. Thus, our findings provide circumstantial evidence that photoperiodic cues are stronger “pull” pressures than extreme temperatures are “push” pressures in late‐winter to early‐spring for waterfowl (Gwinner, 2008).

Lesser Snow Goose and Greater White‐fronted Goose have greater body reserves and thermal inertia than smaller‐bodied ducks and we presumed would withstand extreme temperatures for longer periods of time (Huey et al., 2012; Massey et al., 2020). However, large‐bodied waterfowl (i.e., geese), which employ grubbing and browsing foraging strategies, appeared to be more sensitive to the ECE than small‐bodied dabbling ducks. More pronounced movement of geese were likely related to their grubbing and browsing foraging strategies (Baldassarre, 2014). Snow and ice associated with winter ECEs makes agricultural and other food resources inaccessible and thus geese may need to move to more hospitable environments. Even in the Atlantic Flyway, where the ECE was less severe, resources in coastal wetlands were likely negatively affected forcing geese to move southward and inland in search of more accessible food resources. Moreover, the abundance of agricultural resources (e.g., rice, corn, winter wheat, etc.) in the Mississippi and Central Flyways may have lessened negative effects of the ECE and allowed geese to withstand severely lower temperatures at their wintering latitudes. Additionally, wetland obligate species had appreciable southward distribution shifts during Februarys with severely cold temperatures. Collectively, waterfowl sensitivity to extreme winter ECEs may be a function of specialized foraging strategies (i.e., wetland obligate and grubbing) and the ECEs effect on resource availability.

Overall, our results suggested that ECEs likely alter waterfowl distributions but not in a consistent manner across species or flyways. Cohen et al. (2021) found considerable species‐specific variation across 41 bird species and reported that in general, waterbirds were less sensitive to the mid‐winter polar vortex disruption than most small‐bodied passerines which is likely a result of their foraging life histories (e.g., granivory and insectivory) and body sizes (Cohen et al., 2021). They found that waterbirds increased in occurrence but decreased in abundance, which they interpreted as flocking behavior, making them more conspicuous to eBird users (occurrence increase), but simultaneously suggesting movement away from the affected area (abundance decrease). Although we did not test changes in occurrence and abundance directly, it is likely that similar phenomena occurred in response to polar vortex disruptions in our study; flocking behavior could explain why wetland obligate dabbling duck species were able to persist at latitudes with frozen‐over wetlands. Additionally, our GPS‐telemetry revealed that most Mallards stayed in their wintering area, also suggesting aggregation to keep water unfrozen or habitat switching during extreme cold weather (Jorde et al., 1984; Longcore & Gibbs, 1988; Shilov, 1968). However, specific evaluation of these phenomena were beyond the scope of our study. Nevertheless, more cross‐taxa telemetry studies are needed to examine individual behaviors that enable waterfowl to cope with such harsh temperatures.

Previous studies demonstrated that birds use local cues correlated with climate (e.g., green‐up, day‐length) to predict conditions at distant sites, which in turn dictates phenology across important life history stages (Bauer et al., 2008). Winter ECEs are forecast to increase in frequency, severity, and occur later in winter. These events have potential to misalign with important annual cycle events of waterfowl and other migratory birds (Casson et al., 2019; Cohen et al., 2014). Indeed, our analyses revealed that northward distribution shifts—especially in grubbing geese and wetland obligate dabbling ducks—were stunted or shifting southward as a result of severe late‐winter weather. Thus, spring migration and/or breeding phenological mismatches may result if photoperiodic cues do not reliably predict future conditions at stopover sites or on the breeding grounds (Helm & Gwinner, 2005; Marra et al., 2005). This is especially important for capital breeding Arctic‐nesting geese; these birds have shorter breeding windows and must migrate farther than other dabbling ducks. Phenological mismatches where breeding is no longer optimally timed have already been linked to local population declines (Weegman et al., 2022). Arctic amplification is predicted to simultaneously warm the tundra while intensifying and delaying severe winter events across the mid‐continent (Cohen et al., 2020). Therefore, earlier plant phenology in the tundra combined with delayed spring migrations as a result of later and more severe winter ECEs may increase ill‐timed breeding and other life‐history events across Arctic‐nesting species (Ross et al., 2018; Weegman et al., 2022). Such desynchronization could also affect survival, body conditions, or arrival timing to the breeding grounds for both Arctic‐ and prairie‐nesting waterfowl, all of which have potential to lower individual fitness and contribute to local or continental population declines (Devries et al., 2008; Reed et al., 2013; Weegman et al., 2022).

Here, we took advantage of a natural before‐after experiment using continuously collected high‐resolution citizen science to study the effects of an ECE caused by a series of polar vortex disruptions on seven species of wintering waterfowl populations. Our results suggested that waterfowl are generally resilient to extreme winter temperature anomalies and that photoperiodic cues may be stronger “pull” pressures than extreme weather are “push” pressures during late‐winter. Our results also suggested that responses to ECEs are variable among waterfowl species and could be closely related to foraging strategies. Finally, our results demonstrate eBird's potential to monitor population level effects of increasingly frequent and severe ECEs. Future telemetry studies are needed to understand behavioral modifications used to cope with ECEs. With extreme weather forecast to become commonplace, it is critical for researchers and managers to better understand how species respond to ECEs and identify which are most adaptable and which are most vulnerable. This and other demonstrations (e.g., Cohen et al., 2020, 2021) of eBird's utility in measuring climate sensitivity or resilience to ECEs are promising because it costs little while continuously collecting high‐resolution data that can be used to inform ecology, management, and conservation policy (e.g., Ruiz‐Gutierrez et al., 2021).

## AUTHOR CONTRIBUTIONS

MB, OR, and BC conceived the idea. NM and AK conducted analyses. AB, CH, NM, HH, BC, DC, and DO collected GPS telemetry data. OR extracted and organized eBird data. JF and HH provided financial and logistical support for GPS studies. BC, DC, DO, and MB oversaw project administration. NM and OR wrote the manuscript with editorial guidance from all authors. We all approved the final version of the manuscript.

## CONFLICT OF INTEREST

We are unaware of any conflicts of interest.

## Supporting information


Table S1

Figure S1

Figure S2

Figure S3

Figure S4

Figure S5

Figure S6

## Data Availability

Primary data is archived on Dryad at https://doi.org/10.5061/dryad.vx0k6djv9. Additional data and code are archived on Github at https://github.com/nmasto/eBird_manuscript_GCB. Data from eBird may be downloaded at https://ebird.org/data/download.
