# Supplementary material for: Citizen science reveals waterfowl responses to extreme winter weather
Source: Glob Chang Biol. 2022 Jun 16;28(18):5469–79. doi: 10.1111/gcb.16288 (PMC9545755; doi:10.1111/gcb.16288)
Supplement: Supplementary file 1 — Table S1 [file GCB-28-5469-s002.docx]

**SUPPORTING INFORMATION**

Table S1. Models evaluating effects of the February 2021 extreme climatic event (ECE) and severe February temperature (SFT) on continental- and flyway-scale waterfowl distributions, estimated medians in km (β) with associated 95 and 66% credible intervals.

|  |  |  |  |  |  |  |  |  |
| --- | --- | --- | --- | --- | --- | --- | --- | --- |
|  |  |  |  | 95% Credible Intervals | |  | 66% Credible Intervals | |
| Model | Parameter | Effect Size (β) | SD | Lower | Upper |  | Lower | Upper |
| Extreme Climatic Event (ECE) |  |  |  |  |  |  |  |  |
|  |  |  |  |  |  |  |  |  |
| Continental | Intercept | 143.14 | 17.38 | 109.32 | 177.77 |  | 126.62 | 159.41 |
|  | ECE | −84.40 | 60.40 | −200.69 | 36.08 |  | −110.79 | −61.60 |
|  | Grubbing/Browsing | 42.85 | 25.61 | −7.67 | 93.21 |  | 18.50 | 67.17 |
|  | Wetland Obligate | −86.25 | 25.82 | −137.24 | −35.76 |  | −110.79 | −61.60 |
|  | Grub/Browse:ECE | −115.19 | 90.20 | −290.94 | 60.63 |  | −200.69 | −29.21 |
|  | WetObligate:ECE | 45.12 | 91.50 | −134.37 | 222.87 |  | −42.43 | 131.62 |
|  |  |  |  |  |  |  |  |  |
| Atlantic | Intercept | 201.43 | 31.27 | 141.28 | 263.61 |  | 172.23 | 230.62 |
|  | ECE | −82.32 | 106.42 | −290.56 | 129.88 |  | −182.40 | 16.92 |
|  | Grubbing/Browsing | −184.06 | 44.42 | −272.47 | −96.98 |  | −226.58 | −141.55 |
|  | Wetland Obligate | −183.76 | 44.96 | −273.66 | −96.06 |  | −226.25 | −141.42 |
|  | Grub/Browse:ECE | −94.48 | 153.08 | −396.76 | 207.96 |  | −240.99 | 51.67 |
|  | WetObligate:ECE | −23.60 | 154.10 | −327.99 | 274.12 |  | −167.94 | 123.00 |
|  |  |  |  |  |  |  |  |  |
| Mississippi | Intercept | 262.14 | 34.12 | 195.00 | 238.68 |  | 229.40 | 294.39 |
|  | ECE | −177.54 | 119.73 | −410.10 | 55.91 |  | −291.63 | −63.40 |
|  | Grubbing/Browsing | 4.50 | 50.99 | −95.90 | 104.86 |  | −44.02 | 53.21 |
|  | Wetland Obligate | −100.19 | 50.47 | −200.34 | −0.06 |  | −147.60 | −53.48 |
|  | Grub/Browse:ECE | −17.36 | 174.20 | −353.26 | 323.36 |  | −185.15 | 147.58 |
|  | WetObligate:ECE | 70.28 | 173.18 | −268.54 | 407.45 |  | −94.78 | 234.59 |
|  |  |  |  |  |  |  |  |  |
| Central | Intercept | 131.39 | 37.25 | 57.29 | 204.38 |  | 97.64 | 165.80 |
|  | ECE | −59.32 | 128.59 | −312.85 | 193.32 |  | −180.90 | 60.02 |
|  | Grubbing/Browsing | 192.84 | 47.04 | 99.31 | 285.50 |  | 148.78 | 237.27 |
|  | Wetland Obligate | −103.30 | 47.26 | −196.50 | −10.41 |  | −148.00 | −58.55 |
|  | Grub/Browse:ECE | −220.43 | 165.00 | −534.61 | 104.71 |  | −379.39 | −63.40 |
|  | WetObligate:ECE | 105.90 | 165.36 | −221.35 | 431.07 |  | −51.44 | 262.65 |
|  |  |  |  |  |  |  |  |  |
| Pacific | Intercept | −21.92 | 26.06 | −73.58 | 29.19 |  | −46.62 | 2.88 |
|  | ECE | −19.27 | 89.38 | −196.45 | 156.37 |  | −102.82 | 64.16 |
|  | Grubbing/Browsing | 158.50 | 37.88 | 84.80 | 233.24 |  | 122.58 | 194.41 |
|  | Wetland Obligate | 41.84 | 37.72 | −31.50 | 116.09 |  | 6.13 | 77.56 |
|  | Grub/Browse:ECE | −129.43 | 129.67 | −383.42 | 128.65 |  | −253.30 | −6.92 |
|  | WetObligate:ECE | 24.57 | 130.86 | −231.40 | 279.59 |  | −99.38 | 149.48 |
| Severe February Temperatures (SFT) |  |  |  |  |  |  |  |  |
|  |  |  |  |  |  |  |  |  |
| Continental | Intercept | 143.26 | 17.46 | 109.07 | 178.22 |  | 126.59 | 159.81 |
|  | SFW | −42.02 | 38.04 | −116.18 | 32.74 |  | −78.37 | −5.91 |
|  | Grubbing/Browsing | 43.08 | 26.79 | −9.48 | 94.81 |  | 17.24 | 68.48 |
|  | Wetland Obligate | −86.15 | 26.52 | −138.00 | −33.64 |  | −111.51 | −60.57 |
|  | Grub/Browse:SFT | −58.70 | 57.33 | −170.25 | 53.77 |  | −114.31 | −4.37 |
|  | WetObligate:SFT | −70.17 | 57.22 | −181.61 | 41.87 |  | −123.54 | −16.34 |
|  |  |  |  |  |  |  |  |  |

**SUPPLEMENTAL FIGURE CAPTIONS**

Figure S1. Monthly temperature anomalies (ºC) for February 2008–2021 across the conterminous U.S. Segmentation of the U.S. depicts the Pacific, Central, Mississippi, and Atlantic Flyways (left to right). Temperature anomaly data are based on pixelated mean monthly temperatures compared to 30-year averages of historic temperatures globally (Lenssen et al. 2019).

Figure S2. Medians and 66% posterior distributions (shaded) for continental-scale models where distribution shifts (km) between “early” and “late” periods were modeled as a function of the February 2021 ECE (blue) or severe February temperatures (purple), foraging strategies (i.e., grubbing/browsing, wetland obligate, and generalists), and their interaction.

Figure S3. Medians and 66% posterior distributions (shaded) for flyway-scale models where distribution shifts (km) between “early” and “late” periods were modeled as a function of the February 2021 extreme climatic event (ECE; binomial), foraging strategies (i.e., grubbing, wetland obligate, and generalists), and their interaction. Flyways are illustrated by different colors and reference categories for all models were north-south distribution shifts of generalists during “normal” years.

Figure S4. North-south median distribution shifts (km) and 95% credible intervals (CRI) of continental waterfowl populations in response to the February 2021 ECE separated by flyways (rows). Column one represents estimated marginal medians and 95% CRI averaged across species. Column two depicts estimated marginal median distribution shifts and 95% CRIs across foraging strategies during the ECE (blue) and non-ECE years (red). Foraging strategies included generalists (i.e., Mallard, Northern Pintail, and Teal), grubbing/browsing foragers (i.e., Lesser Snow Goose, and White-fronted Goose), and wetland obligate species (Northern Shoveler and Gadwall).

Figure S5. Male and female Mallard (*Anas platyrhynchos*), Northern Pintail (*A. acuta*), Green-winged Teal (*A. crecca*), Gadwall (*Mareca strepera*), and Northern Shoveler (*Spatula clypeata*) landing in a frozen agricultural wetland in northwestern Tennessee, USA, illustrating food limitations for dabbling ducks created by freezing temperatures. Photograph taken by author CJH on 8 February 2021.

Figure S6. Dabbling ducks flocked in open hole in the ice in northwestern Tennessee, USA, illustrating the lack of useable space resulting from the February 2021 extreme climatic event. Photograph taken on 19 February 2021 and authorized for use by S. Bradshaw
