# Supplementary figures and images for: Citizen science reveals waterfowl responses to extreme winter weather
Source: Glob Chang Biol. 2022 Jun 16;28(18):5469–79. doi: 10.1111/gcb.16288 (PMC9545755; doi:10.1111/gcb.16288)

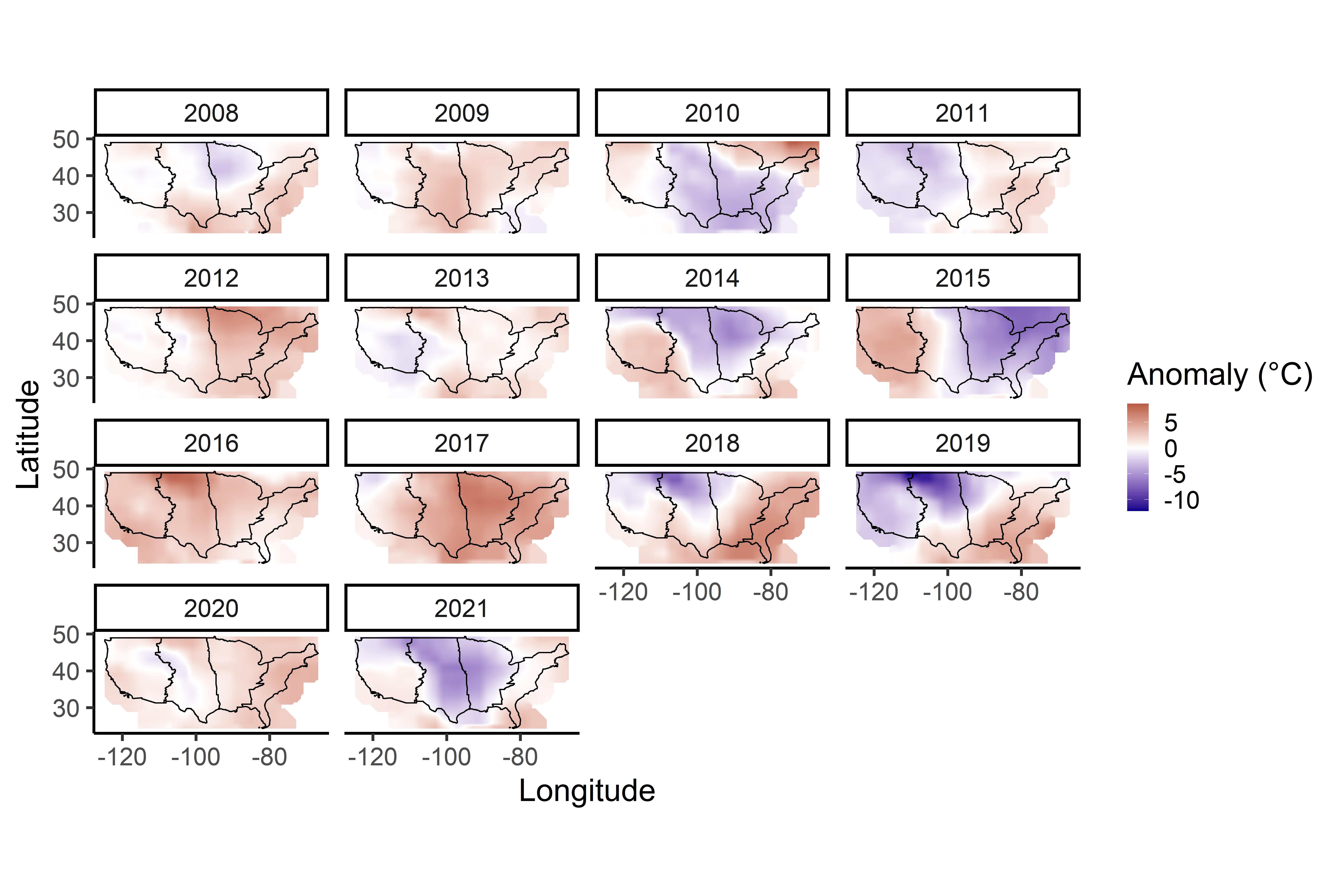

Supplement: Supplementary file 2 — Figure S1 [file GCB-28-5469-s001.jpeg]

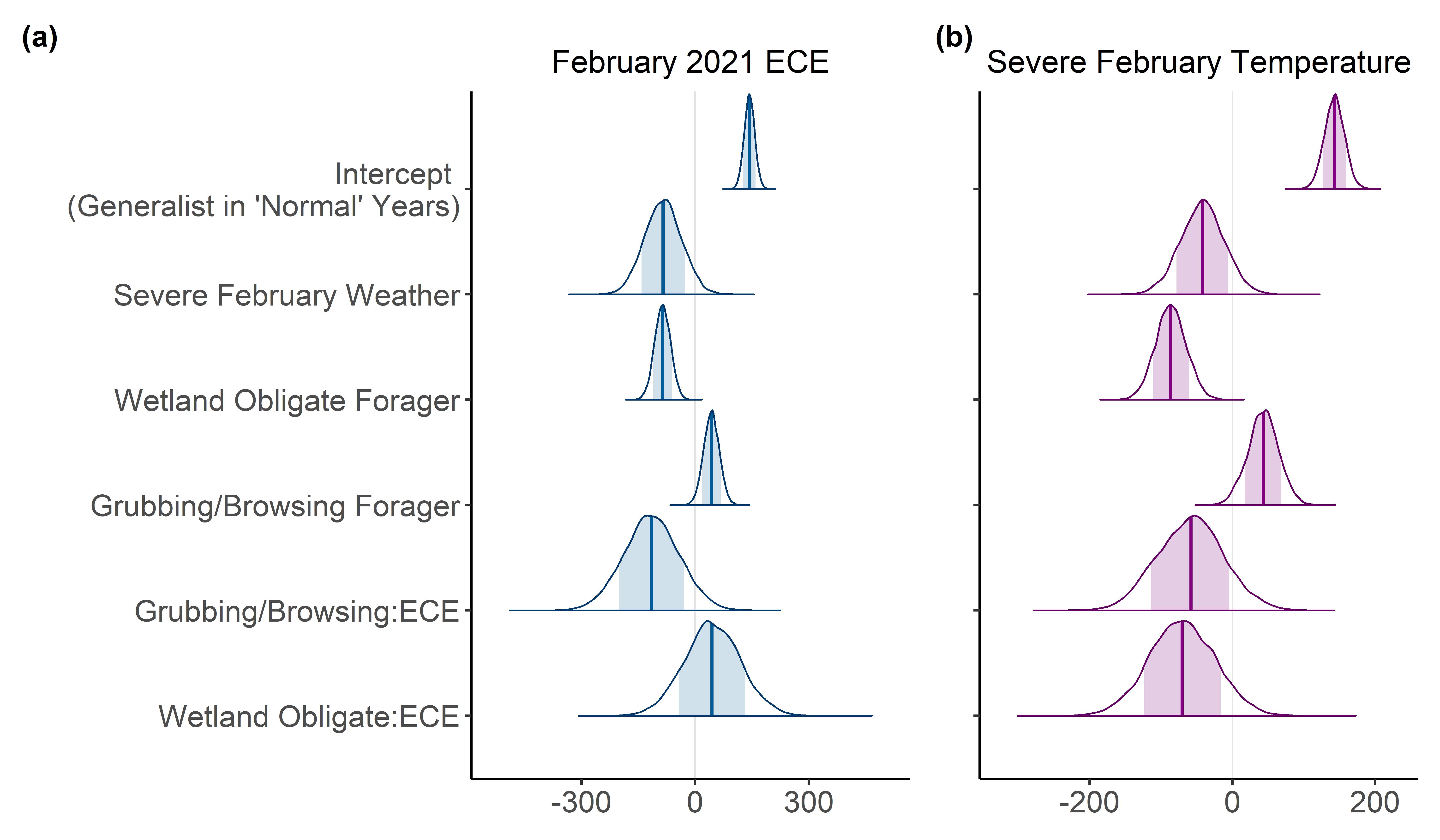

Supplement: Supplementary file 3 — Figure S2 [file GCB-28-5469-s006.jpeg]

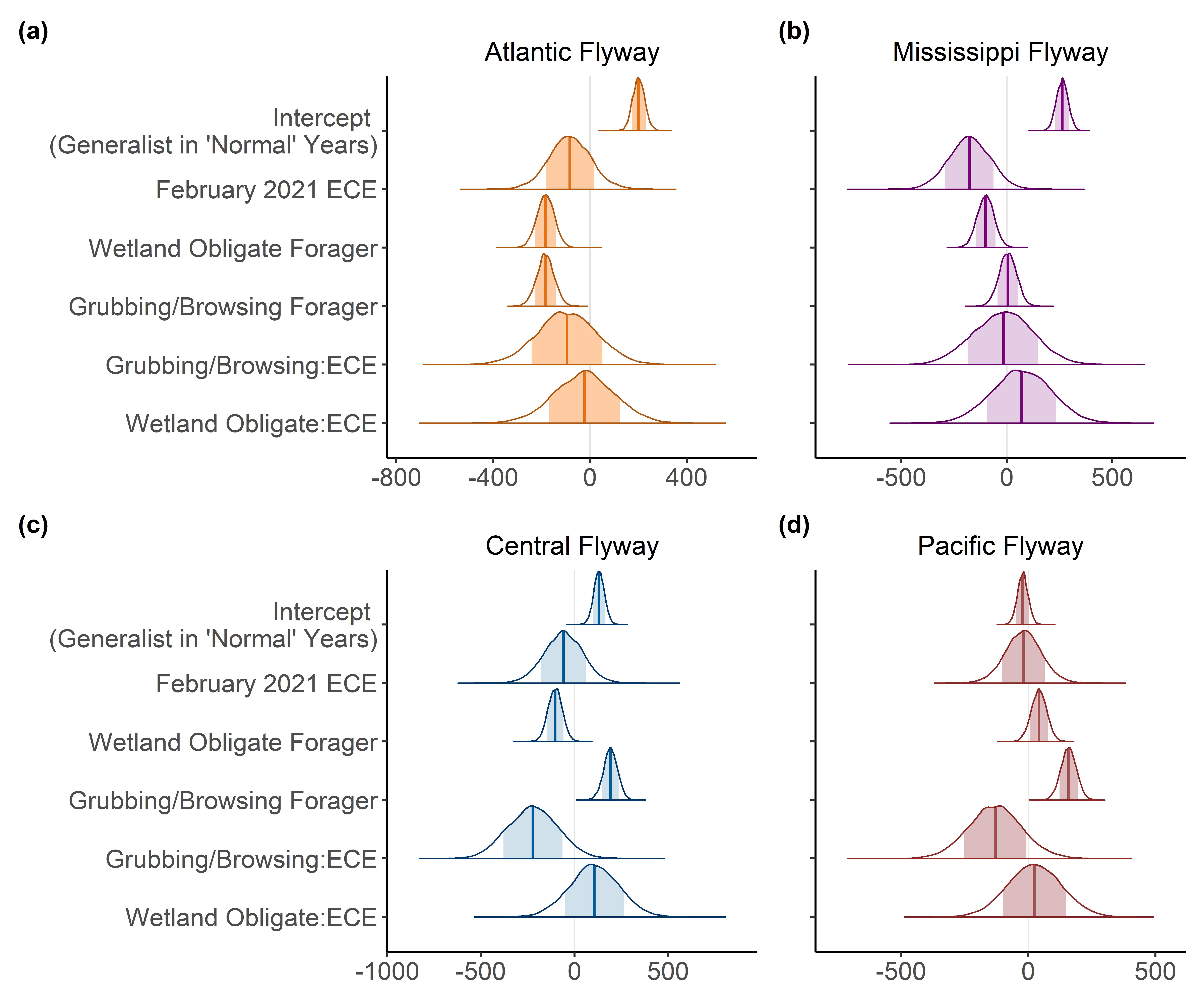

Supplement: Supplementary file 4 — Figure S3 [file GCB-28-5469-s007.jpeg]

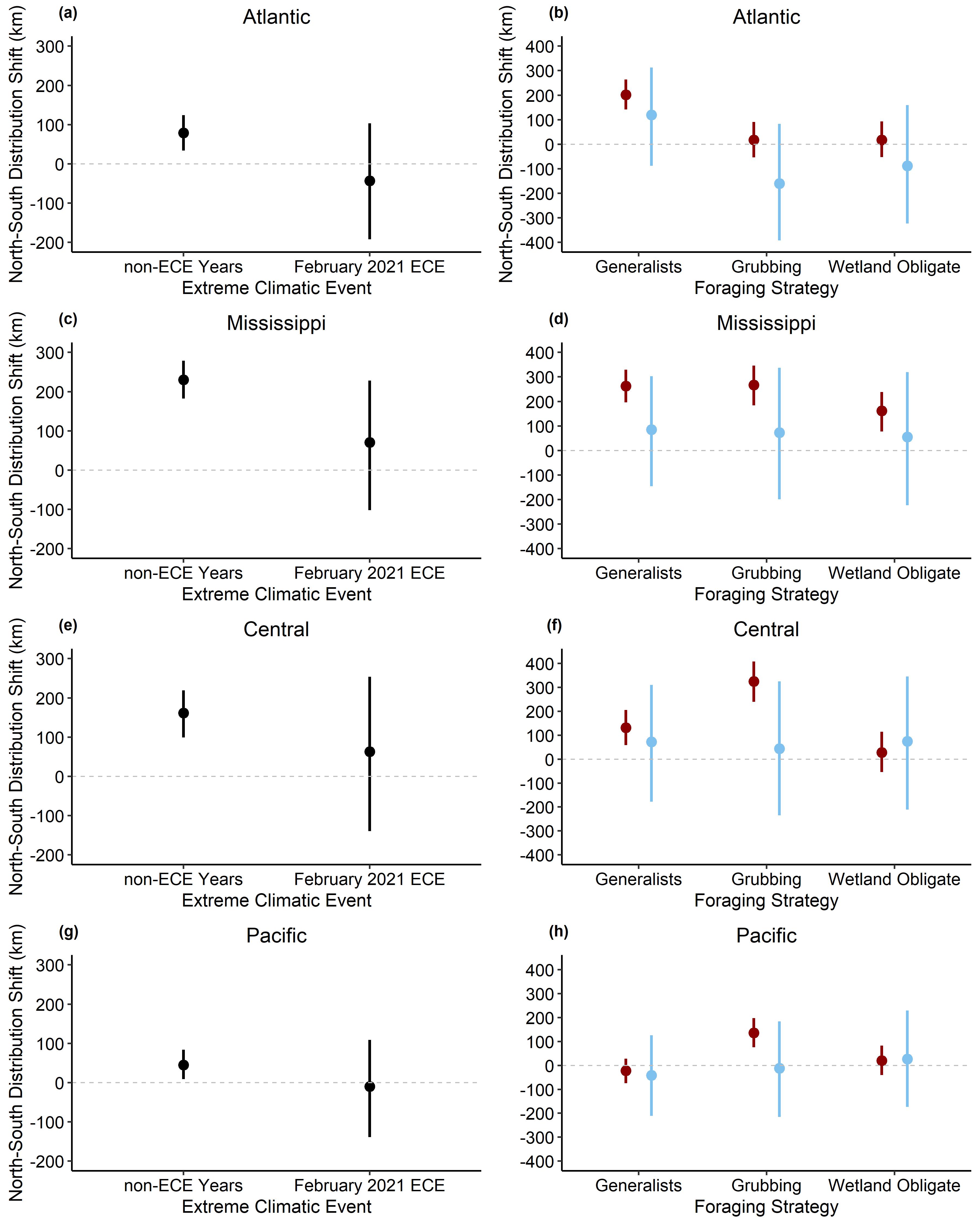

Supplement: Supplementary file 5 — Figure S4 [file GCB-28-5469-s005.jpeg]

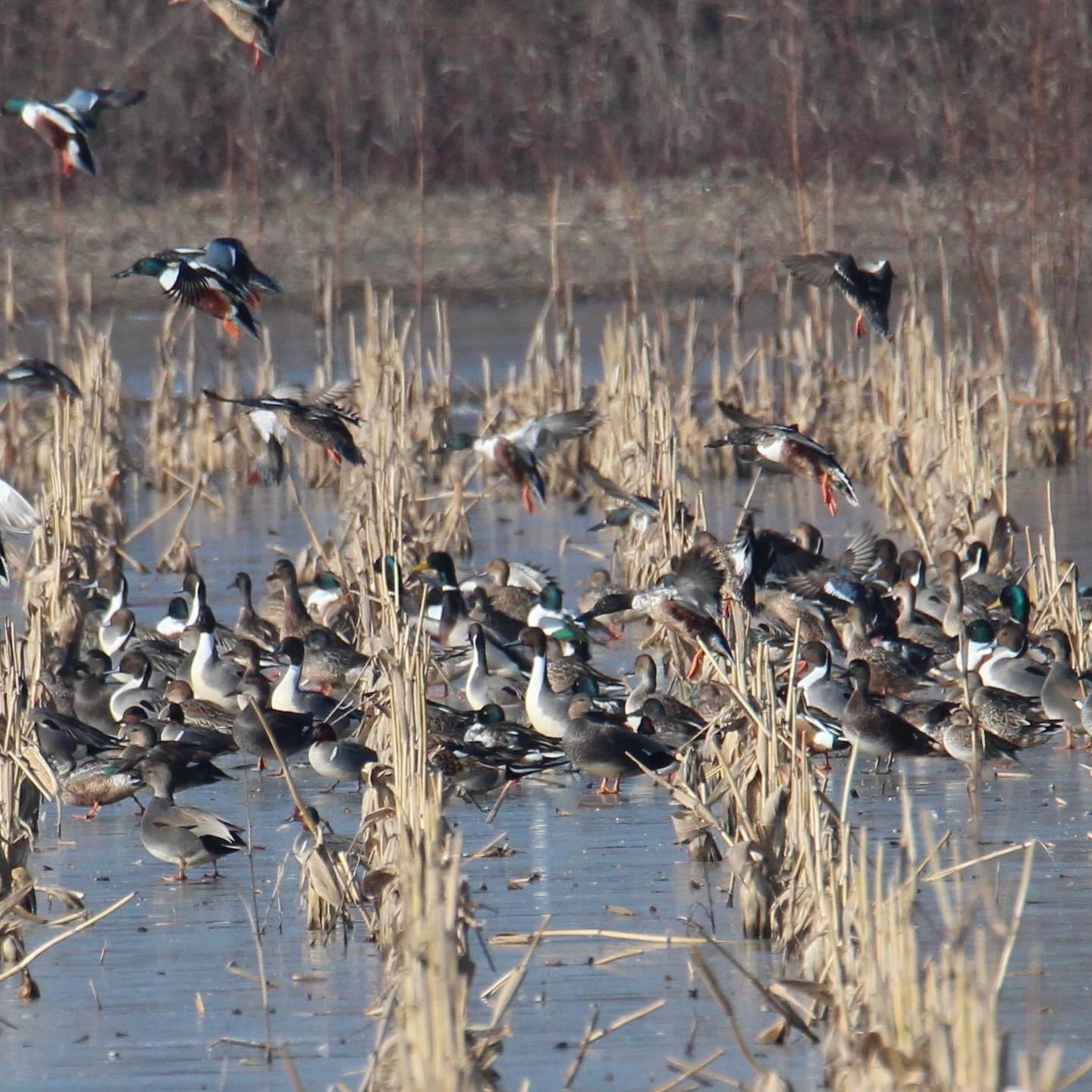

Supplement: Supplementary file 6 — Figure S5 [file GCB-28-5469-s004.JPG]

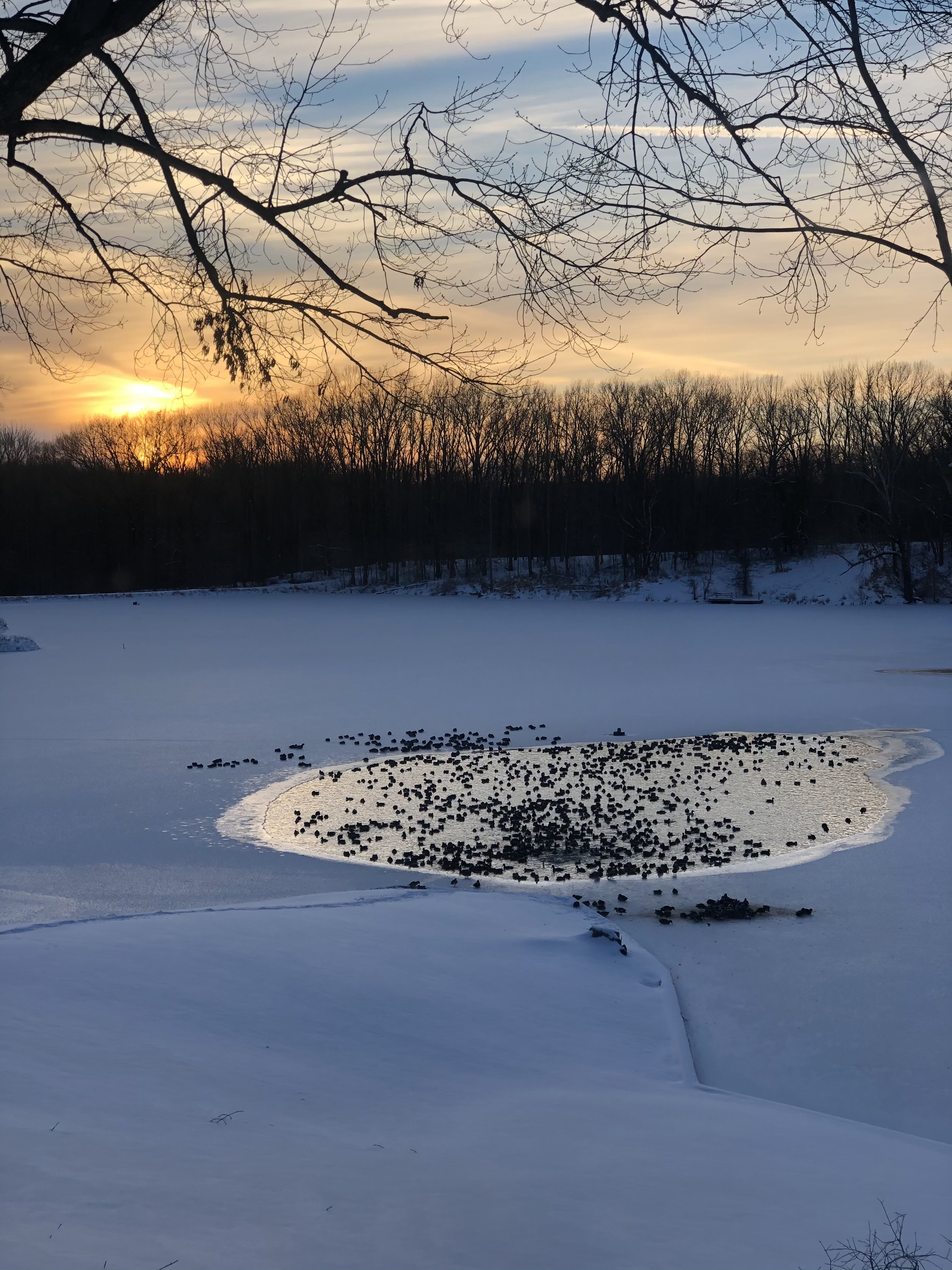

Supplement: Supplementary file 7 — Figure S6 [file GCB-28-5469-s003.jpg]
